# Performance Validation of a Planar Hall Resistance Biosensor through Beta-Amyloid Biomarker

**DOI:** 10.3390/s20020434

**Published:** 2020-01-13

**Authors:** SungJoon Kim, Sri Ramulu Torati, Artem Talantsev, ChangYeop Jeon, SungBae Lee, CheolGi Kim

**Affiliations:** 1 Department of Emerging Material Science, DGIST, Daegu 42988, Korea; becomehero@dgist.ac.kr (S.K.); adt@dgist.ac.kr (A.T.); jco4268@dgist.ac.kr (C.J.); 2 Department of Brain & Cognitive Science, DGIST, Daegu 42988, Korea; sblee@dgist.ac.kr

**Keywords:** planar Hall effect, MR sensor, self-field, β-amyloid, immobilization, detection

## Abstract

Magnetic sensors have great potential for biomedical applications, particularly, detection of magnetically-labeled biomolecules and cells. On the basis of the advantage of the planar Hall effect sensor, which consists of improved thermal stability as compared with other magnetic sensors, we have designed a portable biosensor platform that can detect magnetic labels without applying any external magnetic field. The trilayer sensor, with a composition of Ta (5 nm)/NiFe (10 nm)/Cu (*x* = 0 nm~1.2 nm)/IrMn (10 nm)/Ta (5 nm), was deposited on a silicon wafer using photolithography and a sputtering system, where the optimized sensor sensitivity was 6 μV/(Oe∙mA). The detection of the magnetic label was done by comparing the signals obtained in first harmonic AC mode (1f mode) using an external magnetic field and in the second harmonic AC mode (2f mode) with a self-field generated by current passing through the sensor. In addition, a technique for the β-amyloid biomarker-based antibody-antigen sandwich model was demonstrated for the detection of a series of concentrations of magnetic labels using the self-field mode method, where the signal-to-noise ratio (SNR) was high. The generated self-field was enough to detect an immobilized magnetic tag without an additional external magnetic field. Hence, it could be possible to reduce the device size to use the point-of-care testing using a portable circuit system.

## 1. Introduction

Recently, research on biosensors based on magnetic labels using magnetoresistance has been actively conducted for various biomedical applications [1,2,3]. Simple device configurations of magnetoresistive (MR) biosensors, combined with low noise in concentration measurements of biosamples [4,5], make MR biosensors very promising as in vitro diagnostic devices and point-of-care testing systems [6,7,8]. The largest share of recent works on magnetic biosensors considers giant magnetoresistive (GMR) sensors [9,10] and tunneling magnetoresistive (TMR) sensors [11] as the most promising sensors for biosensor applications. Although the response of these sensors to a magnetic field is not linear, this problem has recently been solved by the design of spin-valve structures with mutually perpendicular magnetization directions in two neighboring ferromagnetic layers [12,13]. In addition, several linearization techniques have been proposed for the GMR and TMR sensors with “conventional” layer sequences, where the magnetizations in active layers are switching between parallel and antiparallel states [14].

**Figure 1:**
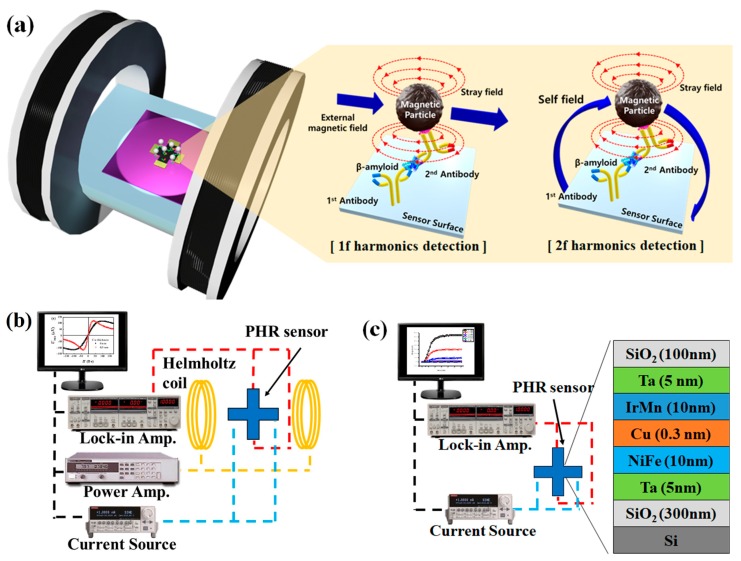
(**a**) Schematics of the planar Hall resistance (PHR) biosensor working principle in 1f and 2f detection mode. (**b**) Block diagram of the experimental setup for the 1f detection mode. (**c**) Block diagram of the experimental setup for the 2f detection mode and schematics of the layer stack of the PHR sensor.

In addition to GMR- and TMR-based magnetic biosensors, several designs have been proposed based on coil magnetometry [15], magnetic resonance imaging [16], Hall effect [17], spin Hall effect [18], magnetoimpedance [19,20,21], anisotropic magnetoresistance [10], and planar Hall effect [22]. As well, several inspired magnetic reception techniques have been proposed in recent works [23]. The advantages of magnetic sensors based on the planar Hall effect (PHE) or planar Hall resistance (PHR) are linearity [24], high signal-to-noise ratio [25], and low thermal drift [26]. As well, the element of a PHR sensor contains fewer layers as compared with the element for a GMR [1] or TMR [27] sensor, which additionally simplifies the fabrication procedure of PHR-based biosensors and reduces their cost. The measurement principle of the PHR-based biosensor is shown in Figure 1a. The biomarker is immobilized on the surface of the sensor through the surface chemistry process. When the biosensor is placed in a solution with magnetically-labeled biomolecules, magnetic tags are absorbed on the surface of the sensor through the binding process between these biomolecules and the biomarkers are immobilized on the sensor surface. The bonded magnetic particles, which are in proximity to the sensor’s surface, generate magnetic stray fields that can be detected by the PHR sensor as a change of its output voltage signal.

As shown in Figure 1b, conventional methods require additional devices such as Helmholtz coils or flux guides to magnetize the magnetic particles [28,29]. The necessity of external magnetic field generators is an obstacle to the development of miniaturized modules due to their physical size limitations. Furthermore, a signal error is generated due to a position error between the external magnetic field generator and the sensor, and thus accurate arrangement between the external magnetic field generator is needed. An alternative scheme for magnetic field generator-free detection of magnetic tags is represented in Figure 1c. In this scheme, the magnetic particles immobilized on the surface of the sensor are magnetized by the sensor’s self-field, which is generated by current passing through the sensor’s current path, and the magnetic particles can be detected without an additionally applied external field. In this work, the PHR sensor with a trilayer structure of the magnetoresistive element [30] was fabricated and used as a biosensor.

We have proposed a measurement method for the development of a biosensor based on this PHR sensor with trilayer structure, by comparing the sensor’s signal measured under alternating current at first and second AC harmonics of this current. We have demonstrated that the detection of magnetic particles, at first AC harmonics, requires the external magnetic field. However, at second AC harmonics, the self-field generated by the sensor’s current path when the AC current is applied is enough to detect particles without an external magnetic field. In addition, the antibody-antigen sandwich model structure was used to detect β-amyloid [31], which is a well- known biomarker of Alzheimer’s disease. Although the detection of β-amyloid using several sensors’ technologies such as optical, surface plasmon resonance, and electrochemical method has already been reported in [32,33,34,35], the detection of β-amyloid using magnetic sensors working in AC-field or AC-current modes has not yet been reported. The serial measurement of a sensor’s signal, for a range of magnetic particle concentrations performed in this work, gives the optimal concentration of magnetic particles at which the resolution of the sensor towards Alzheimer’s detection can be obtained from the measurements on a series of biomarker concentrations. The main goal of this work is the design of a portable biosensor system based on a planar Hall effect that enables the detection of magnetically-labeled biomolecules without an external magnetic field. 

## 2. Materials and Methods

### 2.1. Materials

The streptavidin-coated magnetic nanoparticles (nanomag^®^-D-spio-streptavidin) of mean size 100 nm were purchased from Micromode, Germany. The recombinant rabbit monoclonal antibody (Aβ 1–42) was purchased from Thermo Fisher Scientific, Korea. The [MOAB-2]-biotinylated, mouse monoclonal antibody (Aβ 1–40/42) was purchased from Biosensis, USA. The amyloid-β peptide (1–42) (human) antigen was purchased from Abcam, USA. Phosphate buffered saline (PBS, pH 7.4) was purchased from Bioneer corporation, Korea.

### 2.2. Fabrication of PHR Sensors

In this study, PHR sensors were fabricated using UV lithography and DC magnetron sputtering [36]. The size of the sensor’s active area was 5 μm × 5 μm. The layer composition of the sensor was Ta (5 nm)/NiFe (10 nm)/Cu (*x* = 0 nm~1.2 nm)/IrMn (10 nm)/Ta (5 nm). For biomolecule immobilization, the PHR sensor surface was passivated by a SiO_2_ layer of 100 nm thickness using PECVD.

### 2.3. Characterization of Dynamic Range and Sensitivity of PHR Sensors

In order to optimize the PHR sensor based on a trilayer, a set of sensors were fabricated with a Cu spacer thickness in the range of 0–1.2 nm. The field dependences of the PHR voltage (PHR curves) were recorded at room temperature in a field range from −150 Oe to +150 Oe with a field step of 2 Oe. The driving current passing through the sensor was set at 1mA. On the basis of the peak-to-peak field and voltage of the PHR curve, the sensitivity of the sensor was determined as the slope *dV*_PHR_/*dH* of the PHR curve.

### 2.4. Detection of Magnetic Particles Using PHE Sensors in the 1f and 2f Modes

The magnetic particle measuring method using the PHR sensor includes 1f mode and 2f mode. The 1f mode is a method of inducing and measuring the stray field of magnetic particles through an external magnetic field. In the 2f mode, the magnetic field generated by the sensor itself is used to generate the stray field. 

Generally, the output voltage of the PHR sensor can be written as [37,38]:

(1) $$V_{output}=I\left[S_{0}\cdot \langle H_{eff}\rangle +R_{offset}\right]$$

where *I* is applied current; the ($I\cdot R_{offset}$) product is the sensor’s offset voltage, $S_{0}$; sensor’s sensitivity and the effective field, *H_eff_*, acting on the magnetic biosensor is a sum of three fields, i.e., external magnetic field (*H_ext_*) magnetic stray field (*H_stray_*) from magnetic particles immobilized on the surface of the sensor, and the sensor’s self-field (*H_self_*) generated by current passing through the magnetic sensor:

(2) $$H_{eff}=H_{self}+H_{stray}+H_{ext}$$

When the alternating current

(3) $$I\left(t\right)=I_{0}\cos\left(\omega t\right)$$

the output voltage of PHR sensor can be written as

(4) $$V_{output-1f}=I_{0}\cos\left(\omega t\right)\left[S_{0}\cdot \langle H_{eff}\rangle +R_{offset}\right]$$

Equation (4) can be measured by 1f mode.

The alternating current is applied to the sensor, and the sensor generates alternating self-field

(5) $$H_{self}=\alpha I=\alpha I_{0}\cos\left(\omega t\right)$$

where *ω* is current frequency, *I*_0_ is current amplitude, and *α* is the proportionality coefficient between the current *I* and self-field *H_self_*.

When using self-field (2f mode) the external magnetic field ($H_{ext}$) is not used. Therefore, the effective field ($H_{eff}$) can be reduced as

(6) $$H_{eff}=H_{self}+H_{stray}$$

The stray field by self-field can be written as

(7) $$H_{stray}=I\gamma N_{0}\chi =I_{0}\cos\left(\omega t\right)\gamma N_{0}\chi$$

where χ is the particle magnetic susceptibility, $N_{0}$ is the number of particles, and γ is the constant which depend on the sensor geometry and particles distribution [38].

The output voltage of PHR sensor by self-field can be written as

(8) $$V_{output-2f}=I_{0}\cos\left(\omega t\right)\left[S_{0}\cdot \langle H_{self}+H_{stray}\rangle +R_{offset}\right]$$

To measure the stray field by self-field, the 2f mode is used. The 2f mode uses a twice frequency of reference frequency by the lock-in amplifier. In Equation (8), the offset term can be reduced due to the 1f mode term and only twice frequency terms can be derived.

(9) $$\begin{array}{ll} V_{output-2f} & =I_{0}\cos\left(\omega t\right)\left[S_{0}\cdot \langle H_{self}+H_{stray}\rangle \right]\\ & \;=I_{0}\cos\left(\omega t\right)\left[S_{0}\langle \cdot \alpha I_{0}\cos\left(\omega t\right)+I_{0}\cos\left(\omega t\right)\gamma N_{0}\chi \rangle \right]\\ & \;=I_{0}^{2}\cos^{2}\left(\omega t\right)\left[S_{0}\cdot \langle \alpha +\gamma N_{0}\chi \rangle \right]=\frac{1+\cos\left(2\omega t\right)}{2}I_{0}^{2}\left[S_{0}\cdot \langle \alpha +\gamma N_{0}\chi \rangle \right] \end{array}$$

In Equation (9), let’s consider the dependence term of twice frequency.

(10) $$V_{output-2f}=\frac{1}{2}\cos\left(2\omega t\right)I_{0}^{2}\left[S_{0}\cdot \langle \alpha +\gamma N_{0}\chi \rangle \right]$$

Thus, both $V_{output-1f}$ and $V_{output-2f}$ components of the sensor’s output voltage are linear functions of the number of the particles, *N*_0_, immobilized on the surface of the magnetic biosensor. 

Using a lock-in amplifier, the output voltage of a magnetic biosensor can be detected at selected values of driving and detection frequencies; the $V_{output-1f}$ component (1f mode) is detected when these two frequencies are set at the same value, whereas the $V_{output-2f}$ component (2f mode) is detected when the detection frequency is two times higher than the driving frequency. Although the $V_{output-2f}$ component is of less amplitude than that of the $V_{output-1f}$, the former does not depend on the temperature-sensitive offset voltage, which significantly reduces an error coming from thermal noise. Furthermore, the $V_{output-2f}$ component is excited by the sensor’s self-field and is sensitive only to stray fields of the magnetic particles immobilized on the sensor’s surface and does not depend on the value and polarity of the external magnetic field. One can expect an increased signal-to-noise ratio when the magnetic particle concentration is detected at the 2f mode. In this work, the stray fields of magnetically-labeled particles were detected at *I*_0_ = 4 mA applied current of *ω*/2*π* = 325 Hz driving frequency.

### 2.5. Immobilization of Biomolecules and Magnetic Particles onto the Surface of the Sensor

To demonstrate the suitability as a biosensor using the PHR sensor, β-amyloid, which is a biomarker of Alzheimer’s disease, was immobilized on the surface of the sensor through the antigen-antibody complex. The binding of the antibody on the sensor surface was done through APTES and succinic anhydride linker [39]. Initially, the sensors were given some heat treatment by keeping them in the oven at 100 °C for several minutes and, then, 3 μL of 2% of APTES in DMSO was applied on the sensor surface and kept humidified for 4 hours to make the surface amino group. After successful washing of the sensors, the APTES-modified sensor was incubated with 2 mg/mL succinic anhydride (SA) in THF and 2% triethylamine amino groups for 4 h to convert amine terminal groups to carboxyl groups. Then, freshly prepared 3 μL of 4 mM solution of EDC was dispersed on the SA modified sensor followed by 3 μL of 10 mM NHS and kept for 4 hours to activate the NHS ester.

Then, 3 μL of antibody (0.05 mg/mL) was dispensed on the NHS ester-activated sensor and incubated at 4 °C for 4 hours. Then, the sensor was washed 3 times with PBS and 3 μL of the β-amyloid antigen with a concentration of 0.1 mg/mL was dispersed and incubated for 90 min. Then, the sensors were washed with PBS several times and, finally, incubated with 3 μL of biotinylated antibody (0.05 mg/mL) for 90 min. Then, the immobilized antibody-antigen sandwich complex on the sensor surface was washed with PBS several times and used for the detection of streptavidin-coated magnetic nanoparticles. 

## 3. Results

### 3.1. Optimization of the Layer Composition of the PHR Sensor

**Figure 2:**
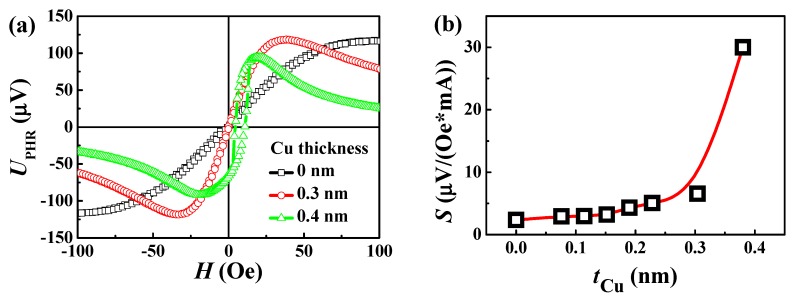
(**a**) PHR curves for the sensors based on varying Cu spacer thickness and (**b**) sensitivity on effective thickness of the Cu spacer layer.

The sensitivity, operating field range and output voltage of the PHR sensor depend on its layer composition. For the PHR sensors based on ferromagnetic/nonmagnetic/antiferromagnetic trilayers, the operating field range is determined by an exchange bias coming from the uncompensated exchange coupling at the ferro-antiferromagnetic interface. With an increase of the effective thickness of a nonmagnetic spacer, the energy of the interface coupling decreases, which reduces the exchange bias and, as a consequence, narrows the operating field range. However, the variations of the PHR output voltage with the spacer thickness are lower than the variations of exchange bias (Figure 2a). Therefore, with an increase of the spacer thickness, the sensitivity (dV/dH) increases (Figure 2b). The limitation for the sensitivity enhancement by spacer thickness is the hysteretic behavior of the PHR signal, which appears when the exchange bias becomes comparable with the coercivity of the trilayer structure. The largest spacer thickness at which the PHR curve has no hysteresis was *t*_Cu.max_ = 0.3 nm. At higher spacer thicknesses, the sensitivity is higher but the PHR signal has hysteretic behavior, as shown in Figure 2b (green curve). At the spacer thicknesses lower than 0.3 nm, the PHR signal is not hysteretic, but the sensitivity is lower than in the sensor with *t*_Cu_ = 0.3 nm. Therefore, the sensor with *t*_Cu_ = 0.3 nm was selected as the optimized one. Its sensitivity to the applied magnetic field was 6 μV/(Oe∙mA), which is three times higher than the sensitivity of the PHR sensor based on the NiFe(10 nm)/IrMn(10 nm) bilayer without a nonmagnetic spacer. 

### 3.2. Operation of the Optimized PHR Sensor in 1f and 2f Detection Modes

**Figure 3:**
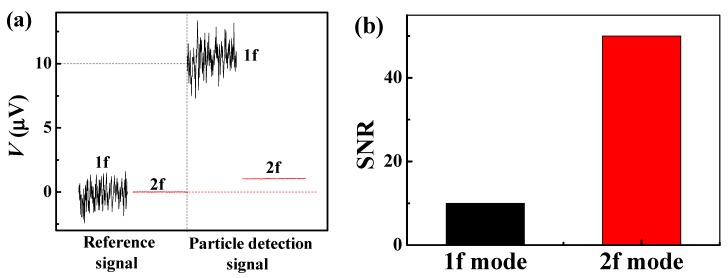
(**a**) Comparison of particle detection signal in the 1f and 2f detection modes and (**b**) comparison of signal-to-noise ratio (SNR) in the 1f and 2f detection modes.

The signals recorded in 1f and 2f modes were compared and analyzed using the same PHR sensor and the same concentration of magnetic nanoparticles. In all experiments (1f and 2f mode), the sensor used the same current source (4 mA, 325 Hz). In the 1f mode, the external magnetic field ($H_{ext}$) used 80 Oe (DC magnetic field) and, in the 2f mode, the external magnetic field was not used. A droplet of $6.9\times 10^{7}$ of “nanomag^®^-D-spio-streptavidin, 100 nm” was deposited onto the sensor. The PHR signal was measured before and after the deposition of the droplet, in both the 1f and 2f detection modes. In the 1f mode, the difference between the PHR output signal before and after the droplet deposition is 10 μV, whereas the corresponding difference of PHR output in the 2f mode is just 1 μV (Figure 3a). By simply comparing the output signals for the 1f and 2f modes, the 1f mode looks better than the 2f mode. However, the noise level in the 1f mode is much higher than the noise level in the 2f mode; the noise level in the 1f mode is 1 μV and the noise level in the 2f mode is ~20 nV. Thus, the signal-to-noise ratios for the 2f mode is higher than the 1f mode (Figure 3b). The advantage is that the 2f mode can be used to detect a signal from magnetic labels without an external magnetic field and without a loss of sensitivity.

### 3.3. Real-Time Detection of β-Amyloid

**Figure 4:**
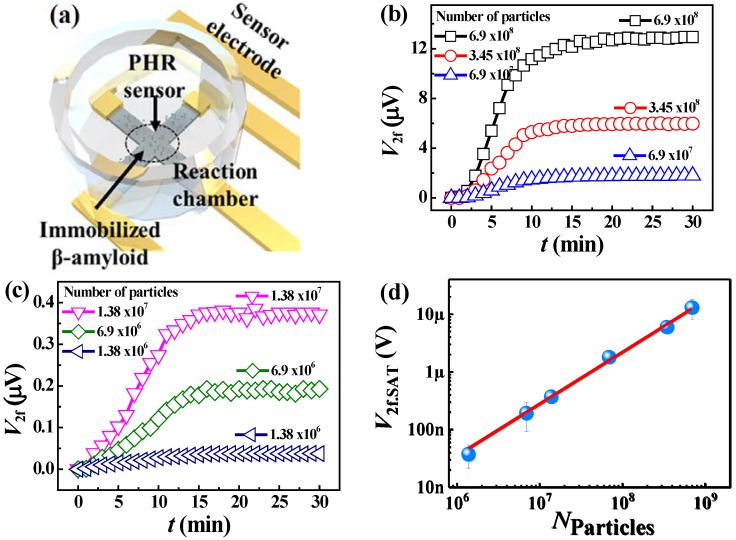
(**a**) Schematic representation of particle detection by PHR sensor. (**b**) Time dependences of the second harmonics PHR voltage *V*_2f_ measured at 3 different numbers of particles in a range from $6.9\times 10^{8}$ to $6.9\times 10^{7}$. (**c**) Time dependences of the second harmonics PHR voltage *V*_2f_, measured at 3 different numbers of particles in a range from $1.38\times 10^{7}$ to $1.38\times 10^{6}$. (**d**) The linear dependence of the saturated value *V*_2f.SAT_ of the second harmonics voltage on the number of particles *N*_Particles_.

The optimized PHR sensor was used for streptavidin-coated magnetic nanoparticles detection through the β-amyloid biomarker. Before dropping the streptavidin-coated magnetic nanoparticles, the sensor surface was immobilized with first antibody followed by β-amyloid antigen and, finally, biotinylated second antibody sandwich complex. The biomarker concentration of β-amyloid was fixed at *C*_β_ = 22 μM. A PDMS well with 2 mm inner diameter was used as a reaction chamber for detection of particles around the sensor area (Figure 4a). The sensor and the electrode were connected under the wall, and the electrode exposed to the outside of the wall was connected to the PCB board by the Au wiring method. The total detection of the magnetic particles was done inside the chamber. Then, a 10 μL droplet of solution with streptavidin-coated magnetic nanoparticles was dropped on to the sensor inside the reaction chamber and the time dependence of the second harmonics PHR signal was recorded, until the PHR signal became saturated. A series of six experiments was performed for different concentrations of streptavidin-coated magnetic nanoparticles from $6.9\times 10^{8}$ to 1.38 $\times 10^{6}$ per 10 μL sample volume (Figure 4b,c). The observed time dependences of the sensor’s output voltage are saturated within 10 to 15 min, which is comparable to the ones observed for GMR sensors [11,13]. The dependence of the saturated PHR output voltage *V*_2f.SAT_ on the concentration of particles (Figure 4d) is linear. The system noise level in the 2f mode is 20 nV. An extrapolation of the dependence of *V*_2f.SAT_ on *N*_Particles_ down to this voltage gives the minimum concentration of particles *N* = 1.38 $\times 10^{6}$ particles per 10 μL detectable by the designed sensor. Although this concentration is higher than the detection limits achieved in several previous works where the external field has been used for magnetizing particles or guiding them to the sensor’s active element, the proposed technique does not require an external magnetic field and has a simplified sensor’s fabrication procedure. Thus, the developed biosensor, based on PHR magnetoresistive element working in 2f mode, can be considered a portable and lower-cost alternative to the existing biosensors for point-of-care diagnostics devices.

## 4. Conclusions

A portable planar Hall effect sensor for detection of magnetically-labeled particles has been designed. The sensitivity of the sensor has been optimized by the introduction of a nonmagnetic spacer layer. The optimized value of spacer thickness is 0.3 nm. At this spacer thickness, the sensor’s sensitivity is higher than the one without the spacer, whereas the field dependence of the signal is still not hysteretic. The use of second harmonics of the sensor’s signal results in an increase of signal-to-noise ratio by more than one order of magnitude, which makes it possible to detect magnetic labels attached to the sensor using only “self-field” generated from current passing through the sensor, without an external magnetic field. The unnecessity of the external magnetic field for the sensor’s operation reduces the size of an end-product device where the sensor is used. The sensor’s performance has been demonstrated by detection of magnetic labels through β-amyloid biomarker. The use of selective detection of magnetic labels to the surface of the sensor was successfully done through the bonding via the β-amyloid biomarker. Although in this study, we used only one concentration of β-amyloid biomarker to validate the sensor performance for the detection of magnetic particles, it is possible to identify the resolution of the sensor by detecting a series of biomarker concentrations. In addition, we believe that it would be useful to use the proposed sensor technology for the quantification of the biomarker in clinical samples.
